# Evidence gaps and challenges in maintaining and increasing vaccine uptake: A Delphi survey with Australian stakeholders

**DOI:** 10.1002/hpja.875

**Published:** 2024-06-03

**Authors:** Penelope Robinson, Chris Degeling, Kerrie Wiley, Stacy Carter, Julie Leask

**Affiliations:** ^1^ Sydney School of Public Health, Faculty of Medicine and Health The University of Sydney Sydney New South Wales Australia; ^2^ Australian Centre for Health Engagement, Evidence, and Values (ACHEEV), School of Health and Society University of Wollongong Wollongong New South Wales Australia

**Keywords:** behavioural research, Delphi method, immunisation, infectious disease, vaccines

## Abstract

**Issue Addressed:**

Increasing and maintaining vaccination uptake is crucial for preventing and managing infectious diseases. In the context of the post‐coronavirus disease 2019 (COVID‐19) pandemic landscape, this paper examines the perceptions of immunisation implementers and policymakers to uncover the challenges and evidence gaps in routine immunisation efforts.

**Methods:**

We conducted an online two‐round modified Delphi survey with immunisation experts, senior public servants, policymakers, policy advisory groups, and representatives from peak bodies from across Australia. We asked respondents to outline what they see as the greatest challenges to increasing and maintaining uptake of recommended vaccines in Australia; the most difficult aspects of their work in vaccination; the largest evidence gaps in vaccine uptake; and the kinds of social and behavioural research they would like to see prioritised.

**Results:**

The two most important challenges for increasing and maintaining vaccine uptake were effectively communicating the benefits of vaccines to parents and the public and ensuring accessible and affordable vaccination services. Participants strongly agreed that ‘communication about the importance of vaccination’ was the most difficult aspect of their work. Consistently important was the need to better engage specific population groups, such as culturally and linguistically diverse people, pregnant people, at risk cohorts, and health care providers. Social and behavioural research about ‘how to effectively address hesitancy’ was ranked highly among participants.

**Conclusions:**

Findings from this project help provide an understanding of the behavioural, social, ethical, and policy knowledge needs for immunisation policy and implementation in Australia. To respond to vaccine challenges, increase coverage and build public trust in vaccination, policymakers and governments should incorporate social research into vaccination programmes.

**So What?:**

Australia is preparing to launch a Centre for Disease Control. This study demonstrates the importance of integrating social, behavioural, ethical, and policy research into the fabric of this new enterprise. It underlines the need to capacity‐build a workforce able to deliver high‐quality research in these areas, address the needs of immunisation implementers and policymakers, and achieve good outcomes for Australians.

## 1 INTRODUCTION

High levels of vaccine uptake are important for controlling infectious diseases. Coronavirus disease 2019 (COVID‐19) had an impact on vaccine uptake of non‐COVID vaccines and there is risk of a global resurgence of vaccine‐preventable diseases.^1^, ^2^, ^3^ Global vaccination coverage declined in 2021–2022 due to the COVID‐19 pandemic^4^, ^5^ and in Australia, there has been a decline in the public confidence of childhood vaccines since 2017.^6^ Vaccination coverage in Australia has declined in the last 3 years. Routine vaccine coverage has dropped for 12 month old children from 94.85% to 93.26% between 2020 and 2023.^7^, ^8^ Influenza vaccine coverage declined from 46% to 27% in children <5 years between 2020 and 2023.^9^

Although there is good epidemiological evidence that vaccination rates have declined since 2019 and they have also become less equitable, it is less clear how to address this decline in the unique post‐pandemic context and what knowledge is needed to support such actions. Through COVID‐19, high‐income countries like Australia have seen a decline in vaccine confidence and coverage. Inequities in vaccination coverage rapidly arose as the COVID‐19 vaccine was rolled‐out: coverage was between 7% and 26% lower in Aboriginal and Torres Strait Islander communities than in the overall population in 2021.^10^ Parental support for routine vaccination dropped from 93% to 84% between 2017 and 2022.^11^

It is essential that research to guide public health action is informed by knowledge users to maximise relevance and impact. Those who are working on the front‐line of vaccination are best placed to reflect on current gaps and priorities. We conducted a modified Delphi survey with immunisation experts, senior public servants, policymakers, policy advisory groups, and representatives from peak bodies across Australia, to inform and refine priority research areas to support vaccination uptake. We invited participants to tell us about their experience intervening to maintain and increase vaccine uptake, with a focus on evidence gaps and the sorts of social and behavioural research they would like access to in their work on immunisation.

## 2 METHODS

A Delphi survey is a method used to create commentaries on a specific topic over multiple rounds, with the aim of building consensus across experts. Survey data are collected from individuals in the first round, and then analysed and presented anonymously to the group in subsequent rounds to elicit further responses.^12^ Delphi surveys have been successfully used to explore a range of health topics,^13^, ^14^, ^15^, ^16^, ^17^, ^18^, ^19^ providing a way to examine topics with a range of stakeholders and experts. We conducted a two‐round modified Delphi survey, administered using the Qualtrics survey website.^20^ This study was approved by the University of Sydney Human Research Ethics Committee (2022/371). Round 1 was conducted in August 2022 and Round 2 was conducted in September 2022. The first page of the survey included a Participant Information Statement. If participants consented to take part, they indicated their agreement by clicking Yes which took them to the survey questions. Return of the completed survey was accepted as informed written consent.

To reveal broad insights, we invited participants from a variety of roles across Australia, including members of policy advisory groups, senior public servants in each state/territory, peak bodies, research organisations, and advocacy groups. We contacted potential participants via publicly listed email addresses to invite them to take part. Two email reminders for each Round of the survey were sent to encourage participation. In Round 1, we asked four open‐ended questions and demographic questions about role, organisation, location, and number of years working in the field. The questions were developed after discussion between the authors. We, as social scientists, were seeking to reset research directions post‐COVID‐19 and guide our work. Over several teleconferences, the authors jointly raised four topics we sought to explore and worked together to refine the related questions.

In both rounds, the survey centred on four main questions:

1. From your perspective, what are the greatest challenges to increasing and maintaining uptake of recommended vaccines in Australia?
2. Think about the most difficult aspect of your work that involves vaccination: What is most challenging and why?
3. What are the largest evidence gaps in vaccine uptake?
4. What social and behavioural research do you wish you had access to in your work in vaccination?

The Delphi method involves a structured communication technique that includes a systematic interaction with a panel of experts. The experts answer questionnaires in two or more rounds. After each round, participants are provided with an anonymised summary of the previous round. Then experts are encouraged to revise their earlier answers considering the replies of other members of their panel. Delphi surveys are a research method that can assess the extent of expert agreement on contentious issues and resolve disagreement and drive towards expert consensus. Modified Delphi surveys are appropriate for research problems that cannot be resolved by technical or objective analysis but can benefit from subjective judgements on a collective basis. We were not aiming to force consensus with this approach. Rather, we were wanting to see how participants ranked each of the topics, to get a clearer sense of research priorities and evidence gaps. Therefore, we employed a modified technique that allowed participants to explain their views.

We used a stratified sample that identified a broad range of relevant stakeholder groups. We identified potential participants from those groups from our knowledge of the field, websites and invited those with publicly listed email addresses. In a Delphi survey, the sample size is determined by participant uptake. Identifying participants was not about reaching a particular statistical sample size, but rather, identifying professionals with pertinent expertise for our research questions. As Wilkes^17^ outlines, Delphi surveys commonly use convenience samples with the expertise of the panel usually being more critical than the sample size. The findings from Modified Delphi techniques are generated by a group of experts through individual reflection and iterative exchanges of knowledge and informed opinion.

Responses were analysed qualitatively and coded thematically by two researchers (PR and CD). PR conducted an initial review of the themes, by examining the qualitative responses and grouping similar topics together. Other authors (CD and JL) reviewed a sample of the responses to ensure consistency of the coding. Next, we compiled lists of the five or six most common responses for each question, so that they could be presented to participants in Round 2. In Round 2, participants were asked to assess and respond to an anonymised summary of the Round 1 responses (see Supplementary material S1). Respondents ranked the challenges and topics raised in Round 1 in order of importance. We scored each of the topics, with five for first‐ranked responses through to one for lowest ranked responses.

## 3 RESULTS

For both rounds of the survey, a panel of 61 people was invited to participate. Round 1 had 22 participants return a completed survey and Round 2 had 21 participants. In Table 1, the most common responses from Round 1 are listed in order from most important to least important, according to the participants' rankings in Round 2. To provide an aggregate ranking of the priorities or level of agreement for each question, we assigned a score to each topic according to its ranking. Highest ranked responses were given a maximum score and total scores were summed for each topic. The totals in the right column (Table 1) represent the sum of the scores assigned to each ranking.

**TABLE 1:** Scored totals for each survey question (Round 2).

	Scored totals
**Most important challenges for increasing and maintaining vaccine uptake**	
Effectively communicating the benefits to parents and the public	113
Accessible and affordable vaccine services	111
Vaccine fatigue	93
Loss of public trust/increase in hesitancy	88
Ensuring providers are educated/up‐to‐date	85
Misinformation/anti‐vaccination movement	66
Complexity/inefficiency of schedules and programmes	60
**Level of agreement with most difficult aspect in your work**	
Communication about the importance of vaccination	176
Inadequate infrastructure/reporting/IT systems for vaccine programmes	129
Not enough vaccine providers/Workforce barriers/Under‐resourcing	129
Training and education for providers	124
**Largest evidence gaps in vaccine uptake**	
Best ways to improve vaccine uptake in certain groups (e.g., culturally and linguistically diverse, pregnant women, Aboriginal and Torres Strait Islanders)	63
Improving vaccine data systems and data collection	58
Addressing vaccine hesitancy	57
Adult vaccination uptake	42
**Importance of social and behavioural research topics**	
Research about how to effectively address hesitancy	167
Research on vaccine attitudes of the public/consumers	164
Research on target groups: adolescent, disability, adults, occupational groups	160
Research into the attitudes of health care providers	158

### 3.1 Challenges for increasing and maintaining vaccine uptake

The two most important challenges for increasing and maintaining vaccine uptake (Q1) were *effectively communicating the benefits of vaccines to parents and the public* and ensuring *accessible and affordable vaccine services*. Participants in the survey also emphasised the loss of public trust in vaccines, since COVID‐19, a sense of what they termed ‘vaccine fatigue’ among the population, and an increased sense of vaccine hesitancy. When asked to explain their reasoning for their choices, participants emphasised the importance of good communication for increasing and maintaining vaccine coverage. For instance:

Communication is key, if we don't get that right then we will not succeed.

Many consumers will take advice from healthcare providers so how messages and advice are delivered, is essential.

Frank communication about the limits to what vaccines can achieve is also challenging for some vaccines (e.g. flu) where we need to promote realistic expectations without undermining confidence in the particular or other vaccines.

The affordability and accessibility of vaccine services were also ranked highly by participants:

Accessibility is a huge issue. Most vaccines on NIP and COVID vaccines can readily given by nurses but need to align with a doctor appointment for billing purposes.

Cost is an issue for some people, especially if there is no bulk billing (BB) for the vax appointment. Some GPs are no longer BB pensioners.

In discussing vaccination uptake, several participants used the term ‘vaccine fatigue’ and stressed the importance of counteracting what they saw as a rise in the mistrust in vaccines. For example:

The order was chosen because the fundamentals of providing the service are critical, followed by how we deal with vaccine fatigue and lack of trust/misinformation, including the communication and health practitioner training required.

We are definitely seeing vaccine fatigue, so being able to effectively communicate is important – especially because vaccination schedules are complex.

Managing misinformation and mistrust in vaccines was viewed as a significant challenge by several participants, with some blaming the COVID‐19 pandemic as a reason for the rise in hesitancy and lack of trust, and some suggesting that confusing Government messaging is a factor. For example:

The COVID antivax messaging was unprecedented AND the constant changing of messaging coming from the Gov also eroded people's trust with many concerns about truth in messaging vs Gov propaganda (whether that perception was valid or not).

Vaccines have come out of the COVID‐19 pandemic with a bit of a jaded reputation. Are people getting sick and tired of being told they must have this vaccine? I think so.

### 3.2 Most difficult aspects of work in vaccination

We asked participants to indicate their level of agreement with the most difficult aspects of working in vaccination. ‘Communication about the importance of vaccination’ scored the highest, with the ‘training and education’ and ‘inadequate infrastructure/reporting’ ranking equal second. Ranked lowest were issues of resourcing or workforce barriers such as a lack of vaccination providers. The bottom three were ranked similarly difficult, with communication ranked the most difficult.

Participants in Round 2 strongly agreed that ‘communication about the importance of vaccination’ was the most difficult aspect of working in vaccination. Some discussed the lack of sociological and anthropological research used in the development of vaccine communication strategies. For instance:

Communication is the foundational piece to get right. It has been largely driven by ‘campaign comms’ through third party ‘brand/advertising‐focused’ agencies, without co‐design and without significant socio‐anthropological or public health input.

Participants scored ‘not enough vaccine providers’ and ‘inadequate infrastructure’ equally in second place. Limitations with the existing vaccine infrastructure were mentioned by several participants, including issues such as problems with accessing vaccination data, or the Australian Immunisation Register (AIR) not being up to date. For example:

Locally, we could benefit from better links between vax data and our communicable disease data. Useful, but not highest or most immediate priority.

Education for immunisation providers was ranked lowest, with some participants raising the importance of vaccine hesitancy training:

Training is complex – we have great access to training to be qualified but not much more on dealing with hesitancy and recalling complex clients. There are heaps of vaccinators, however difficult to maintain effective models of care after the pandemic.

### 3.3 Largest evidence gaps in vaccine uptake

Consistently important to participants was the need to better engage specific population groups, such as culturally and linguistically diverse, pregnant women, Aboriginal and Torres Strait Islander communities, at risk cohorts and health care providers. Participants also stressed the importance of improving vaccine data systems and addressing hesitancy.

Several participants mentioned the need for stronger social and behavioural research to inform these key topics and to help improve vaccine uptake. Participants in Round 2 suggested that more research focusing on vaccine uptake for specific population groups was needed, particularly on strategies for better engagement and less of a ‘one‐size‐fits‐all’ approach. For example:

The diverse engagement and communication needs have been long‐neglected and contribute to inequity in cohorts who already suffer multi‐faceted inequity of outcomes.

Reaching everyone is critically important, one message doesn't reach all.

Another significant evidence gap ranked highly by participants, was the need for improved vaccine infrastructure and data collection systems. For instance,

The AIR (Australian Immunisation Register) does need a major overhaul. Reports need to be more tailored. Data often doesn't match. There are certain aspects of reporting that need attention. For example if dose 2 and 3 of Hexa have been recorded and dose 1 hasn't been, the AIR does not see this is being overdue – there are many issues such as this one.

If the data collection system enabled better patient/vaccination call‐back solutions for practitioners, I think there could be a big improvement.

Some mentioned the importance of improving data systems, while also acknowledging the need for qualitative data:

Through improving data collection and reporting we could improve understanding in a lot of areas, but there does also need to be qualitative work to understand the barriers.

Qualitative data about the social aspects of vaccine behaviour was mentioned in relation to improving communications. Several participants suggested the need for better targeted communication strategies to ensure equitable outcomes and to avoid stigmatisation of certain populations. For example:

Messaging to certain groups needs to be tailored to ensure it is culturally appropriate and inclusive and the communities are actively engaged. The was a lot of negative press and victim blaming by the media. The media needs to be part of the big picture for future comms, and poor behaviour and misreporting needs to be called by Gov.

At the moment we fly blind, which allows health to make assumptions. This needs to change and we need to have a better and more strengths‐based way of reviewing our population.

Ranked last out of the four, several participants discussed the relative lack of research into improving adult vaccine uptake. For example:

Adult vaccination uptake. This is a difficult group to assess for coverage. Adults on a whole do not appear to be concerned whether they are up to date of not unless it is required for employment or travel purposes. Again, should be part of the conversation with health care providers.

### 3.4 Importance of these social and behavioural research topics

Participants were asked to express their level of agreement with four areas of social and behavioural research that were raised in Round 1. When scored, ‘research about how to effectively address hesitancy’ was in the top position, but they were fairly evenly spread. The experts who completed the survey generally ranked all four topics as important or extremely important, with all four research areas receiving a ranking of 8 or above (i.e., extremely important).

Research about how to address vaccine hesitancy was ranked most important, with participants indicating that more complex understandings of hesitancy are required. For example:

Means to address hesitancy are currently simplistic, generalist and poorly informed by socio‐anthropological insights.

Several participants discussed the need for more research into the attitudes and perceptions of health care workers, especially as they are the source of vaccine information for lots of people:

Evidence suggests that health care providers and their advice is the most effective way to vaccinate and address hesitancy. We need to understand ourselves first.

All are important. Hesitancy a significant challenge. Evidence shows health care providers are influential in providing education to consumers so is important to understand their attitudes.

Some participants wanted to see more detailed and ongoing data about vaccine behaviour and attitudes:

I believe that the NIP and broader vaccination programs would be well‐supported by longitudinal tracking research of vaccination behaviours (levels of support/lack of support etc.) of both the public and health professionals. This could then inform planning and strategies to understand and address barriers to uptake.

A good understanding of consumer attitudes is key to guiding the preparation of information, messaging and marketing of that information to the public.

Finally, it is important to note that we asked participants to indicate from 0 to 10 how important they felt each of the topics were. Nearly all participants rated the four research areas as 8 or above, extremely important:

I think the top 3 issues are equally important – equally ‘very’ to ‘extremely’ important.

Table 2 describes the type of organisation and Table 3, the roles of participants. We did not find any patterns in responses based on the organisation or roles of the participants.

**TABLE 2:** Type of organisation of participants (Round 2).

Type of organisation	Count
State or territory government	11
Professional association	3
Commonwealth government	2
Healthcare	2
Peak body/GO	2
University/research institute	1
Other (please specify)	0
Total	21

**TABLE 3:** Roles of participants (Round 2).

Roles	Count
Policy	13
Programme management/implementation	13
Technical expertise	7
Advocacy	5
Research/academia	3
Statutory and regulatory roles	1
Total^a^	42

^a^ Respondents could choose more than one role that applied to them, thus the total number of roles is higher than the number of participants.

## 4 DISCUSSION

Decisions about vaccination policy are not just a matter for policymakers and government: successful implementation requires cooperation between governments, public health agencies and the community. Increasing and maintaining vaccine uptake also requires cooperation across sectors. Social and behavioural insights into vaccine uptake are crucial for how vaccine programmes are organised, services are delivered, health workers communicate, and public access and confidence are sustained with a focus on equity. Our modified Delphi survey with key immunisation experts, stakeholders, advisory groups and policymakers provides insights that will help prioritise the social research undertaken to support this field and help address challenges to increasing and maintaining vaccine uptake.

Our respondents put communication about vaccination with the public as one of the most important challenges and relatedly, wanted to see more research on how to address vaccine hesitancy. They also highly ranked accessible and affordable vaccination services and specific population groups as needing attention: adolescents, adults in general, people with a disability, and occupational groups. There were evidence gaps in ways to improve uptake in certain groups and better utilising data systems. Workforce needs were also a major theme: respondents nominated the education and training of providers as being important along with issues of under‐resourcing.

This survey was conducted August–September 2022, at a time when Australia was recovering from the COVID‐19 pandemic. Most of our respondents had had intensive involvement in the prior year, delivering COVID vaccines to 95% of the eligible population of Australia. Responses will have been influenced by these challenges and won't necessarily predict needs of the future. Furthermore, only 22 of the original invited panel of 61 took part in the survey, making us unable to give the differences in rankings statistical inference. Nevertheless, the study is useful in informing potential prioritisation of social and behavioural research in immunisation and the scope of areas is sufficient to encompass potential future needs.

Attention to enhancing systems for the collection of data on which population groups most commonly miss out on vaccines needs to be matched by a focus on the specific values and processes that motivate public actions. Key to this is the prioritisation of equity and the institutionalisation of procedures that permit for authentic community involvement in decision making and programme implementation. During the pandemic, public health authorities have attained mixed success at securing public trust and community support for COVID‐19 vaccination. The perceived legitimacy and acceptability of vaccination strategies depend on the extent to which they align with community values.

Respondents also emphasised the loss of public trust, ‘vaccine fatigue’, and increased hesitancy. To respond to these challenges and increase coverage, policymakers and governments must incorporate social research into vaccination programmes. Achieving and maintaining equitable vaccine coverage across the country is important. There continue to be major disparities in COVID‐19 booster coverage that leave specific priority population groups more at risk of severe disease. For example, 10.3% of Aboriginal and Torres Strait Islander people had received a 2023 booster dose.^21^ These challenges and their solutions require high‐quality data from the social sciences. Social and behavioural insights from key communities help implementers and policymakers understand what influences vaccination uptake at individual, community, service and policy levels and how to increase coverage and sustain public trust in vaccine programmes. Targeted campaigns, informed by robust social and behavioural research, can help address disparities in vaccine coverage.

Delphi survey methods have several well‐known limitations, including that the substantive outcomes consist of a set of group intuitions and perspectives, constructed through highly structured social processes of justification among experts.^22^ However, as noted in the introduction, the method is particularly useful for the evaluation of complex problems where the rate of social or technological change exceeds the rate of innovation in governance^23^ as could be argued in a post‐COVID vaccination environment. The initial response to participant invitations was moderate, which was gratifying, given that our invitation was unsolicited. Retention of participants for the subsequent round was also high, and the balance between members of different sectors remained constant, meaning that the risk of selection bias due to participant withdrawal is minimal. Moreover, allowing participants to express their views and comment on each other's interpretation, via open‐ended free text questions, over two survey rounds increased the reliability of the study and improved the validity of the results.

While both rounds of the survey included open‐text fields in which participants could explain their choices further, it is possible we did not capture all potential themes. Probes could have been used in a face‐to‐face survey (instead of online) but we believe we would not have had as many respondents without the convenience of an online survey, in which participants could participate at their convenience. This Delphi survey has captured the perspectives of representatives of expert groups concerned with improving vaccination uptake in Australia.

## 5 CONCLUSION

As Australia prepares for a Centre for Disease Control, embedding social research in government and policymaking is essential. Findings from this project help provide an understanding of the behavioural, social, and policy knowledge needs for vaccination programmes in Australia. The study has contributed to a deeper understanding of the research gaps in this area, highlighting that communication is one of the areas of greatest challenge, and the greatest need for new knowledge, underpinned by rigorous social and behavioural research.

The survey uncovered the primary concerns of stakeholders in the field and identified important priorities for future research. Based on this survey we recommend the following behavioural and social science research priorities: (1) Increasing vaccine equity for priority populations, by identifying the causes of coverage gaps and testing strategies to address them; and (2) Improving vaccination communication by generating insights into how communities, professionals and leaders communicate about vaccines. There is a strong relationship between these challenges and policy processes where dilemmas and trade‐offs are encountered. For this reason, we add an additional priority; (3) Bringing public values into vaccination policy, by investigating the ethical and social dimensions of policymaking.

The findings from this study will contribute to the strategic research planning for researchers and strengthen knowledge exchange through seeing research informed by its end‐users from the start. Social and behavioural research is crucial to improving and maintaining vaccine coverage and for the effective management of infectious diseases. The insights generated by this study demonstrate the importance of social research for effectively responding to disease outbreaks, while also minimising health disparities.

The insights also give guidance on directions in policy and practice that may assist recovery of routine immunisation post‐COVID‐19. Access to primary care has reduced since the pandemic with a reduction in bulk billing in general practice and fewer services taking on new patients, particularly in regional areas.^24^ New models of vaccine provisions are needed, for example, that enable nurse immunisation providers to vaccinate more independently of medical oversight. Aboriginal Health Practitioners and Workers nationally should be enabled to vaccinate with appropriate education. To address the reduction in vaccine confidence, our participants emphasised the importance of communicating the benefits of vaccination and equipping immunisation providers in their vaccination conversations. Many of our participants emphasised the importance of more nuanced communication, whether that be in the clinic or public arena. It was noted that campaign communications occurred under contracts with private agencies without sufficient public health and local community input. The federal government should find ways to engage in more grassroots community perspectives with a focus on poorly reached population groups who experience inequities in access to information, resources, and vaccination. Our participants also emphasised that while the Australian Immunisation Register had many strengths, it also had limitations. This is particularly the case in using data to pinpoint specific population groups where coverage is lower. Currently, this relies on linked data analyses that are not routinely available to local public health units. This availability, if done with sufficient privacy provisions and attention to ethical issues, would enable community initiatives to be better targeted and tailored.

## FUNDING INFORMATION

This study was funded by an NHMRC Investigator Grant.

## CONFLICT OF INTEREST STATEMENT

Julie Leask receives payment from the National Health and Medical Research Council and the TGA for sitting fees and is funded by the NHMRC and WHO. This research was undertaken to inform planning for a collaborative research application. Authors Degeling, Wiley, and Carter also receive NHMRC funding.

## ETHICS STATEMENT

The study was approved by the University of Sydney's Human Research Ethics Committee, approval code: 2022/371.

## Supporting information


**Data S1.** Supporting Information.

## Data Availability

Research data are not shared.
