# Supplementary material for: Evidence gaps and challenges in maintaining and increasing vaccine uptake: A Delphi survey with Australian stakeholders
Source: Health Promot J Austr. 2024 Jun 3;36(1):e875. doi: 10.1002/hpja.875 (PMC11729870; doi:10.1002/hpja.875)
Supplement: Supplementary file 1 — Data S1. Supporting Information. [file HPJA-36-0-s001.docx]

**Supplementary material:**

Summary of dominant themes from Round 1 of the survey, with relevant quotes from each question.

| Themes | Q1 Quotes |
| --- | --- |
| Accessible and affordable vaccine services | “Access to medical/nurse appointments in a timely way.”  “Making vaccinations easy to access and free at point of delivery.” |
| Vaccine fatigue | “There is definitely vaccine fatigue post-COVID”  “Vaccine fatigue - ie. that people are currently being asked to get vaccinated” |
| Misinformation/ anti-vax movement | “program undermined by prevalent misinformation”  “The rise of the antivax movement”  “Social media anti vaccination campaigns. These seem to influence people to sit on the fence so to speak.” |
| Effectively communicating the benefits to parents | “It is also essential to ensure realistic expectations about what vaccines can achieve are effectively communicated.”  “new cohort of parents annually, need to reinvigorate immunisation campaigns” |
| Ensuring providers are educated/up-to-date | “Health professionals not being educated enough on vaccinations and being able to talk to consumers about benefits vs risks of diseases, herd immunity etc |
| Loss of public trust / increase in hesitancy | “People appear to have lost faith in the efficacy of vaccines as they see multiple doses of COVID-19 vaccines required and people still contracting the disease.”  “Public trust and understanding of why this is necessary” |
| Complexity/inefficiency of schedules and programs | “Having different vaccination schedules in each state.”  “Increasing efficiency of the recommended vaccine rollout programs (NIP, flu etc)” |

| Themes | Q2 Quotes |
| --- | --- |
| Communication with the public about importance of vaccination / ensure public health expertise in campaigns | “Communicating messages about vaccination, what are the benefits and risks, trying to communicate to the population who may have different perceptions and experiences.”  “Ensuring public health expertise is the foundation for development of communication and engagement content and strategy” |
| Inadequate infrastructure/ reporting/IT systems | “Rural and remote data and location accuracy- locations ids are highly inaccurate which makes finding out the picture difficult”  “Inadequate IT systems to support mandatory reporting to the AIR especially in public facilities.” |
| Not enough vaccination providers / Workforce barriers | “Not having enough vaccination providers to administer vaccines”  “Workforce barriers eg nursing can play a larger roll (and we have more Accredited Nurse Immunisers in Australia than ever before) but they are constrained by Commonwealth funding models in general practice” |
| Funding / under-resourcing | “under-resourcing of public health education and community development” |
| Training/education for providers | “We also need to remember that there is a constant turnover of staff and new nurses coming into general practice a and PHC so need to make sure training is ongoing in the workplace” |

| Themes | Q3 Quotes |
| --- | --- |
| Best ways to target certain groups (e.g. CALD, pregnant women) | “CALD populations, pregnant women, at-risk populations, delayed vaccinations”  “Understanding the groups unlikely/slow to take up vaccines and what are the specific barriers - more targeted provision in evidence about risks and benefits” |
| Improve data systems / data collection | Data systems in Australia are highly underdeveloped and insufficient to support evidence based decision-making - despite much advocacy for same  “an ongoing data collection monitoring vaccine knowledge and attitudes would serve a range of benefits.” |
| Addressing vaccine hesistancy / confidence | “The most effective ways to address mis- and dis-information on social media and tools to combat the amplification of this through effective media”  “Activities that can decrease vaccine hesitancy” |
| Adult vaccination/uptake | “market/media push for the benefits vaccination for adults” |

| THEMES | Q4 Quotes |
| --- | --- |
| Research on vaccine attitudes of the public/consumers | “See above re monitoring survey. Perhaps a task for the ACDC!: "*Back in Vax*" or "*V A X - its Dynamite*"  “understanding of consumer views of vaccine benefits vs risks of diseases.”  “I would like to see research on what the general public are thinking about vaccines on the whole after the COVID-19 pandemic - how can we restore faith in vaccines?” |
| Studies about how to effectively address hesitancy | “Studies showing what works in encouraging vaccine hesitant people to become vaccinated”  “More in-depth, qualitative research with vaccine hesitant individuals and groups and detailed analysis of challenges in the enabling environment for greater uptake.” |
| Research on target groups: adolescent, disability, adults | “Evidence summaries on the barriers to uptake in different groups and strategies that might have been effective.”  “More information on barriers to adolescent uptake, and what would make them more interested in getting their school vaccines.” |
